# The moderating role of moral disengagement on the relation between bullying and school well-being in adolescents

**DOI:** 10.1186/s40359-025-03832-4

**Published:** 2025-12-13

**Authors:** J. Eilts, J. Wilke

**Affiliations:** Department of Special Needs Education and Rehabilitation, Carl von Ossietzky Universität Oldenburg, Ammerlaender Heerstraße 114-118, Oldenburg, 26129 Germany

**Keywords:** Bullying roles, Moral disengagement, School well-being, Adolescents

## Abstract

**Background:**

In recent years, research on bullying has focused on the association between moral disengagement and bullying behaviors. Additionally, the adverse effects that bullying has on school-level variables have been investigated. However, studies investigating the interplay between these variables are lacking.

**Method:**

Therefore, the present study focuses on the moderating effect of moral disengagement on the association between bullying roles (bully, victim, assistant, outsider, and defender) and school well-being. 216 (53.7% female; *M* = 12.84 years, *SD* = 1.39 years) secondary school students from Bremen and Lower Saxony, Germany, participated in the study from January 2022 until July 2022.

**Results:**

The results highlight the moderating role of moral disengagement in the association between victimization experience and school well-being. According to these findings, the more victimization someone experiences, the worse they feel in school. However, the detachment from moral norms weakens this bond.

**Conclusion:**

The discussion explores the notions of cognitive scripts, reduced moral beliefs, and their potential influence on individuals repeatedly victimized by bullying. The focus is on how these individuals may cope by distancing themselves from moral norms.

## Introduction

### Bullying roles

Bullying is a pervasive problem that affects individuals and is a highly debated topic in society and research [1, 2]. Although there is no uniform definition, bullying generally comprises aggressive behavior intended to harm, intimidate, or control another person [3–5]. Research describes three key characteristics that describe bullying. It is characterized by (1) repeated, (2) intentional mistreatment that involves a (3) power imbalance, with the victim being unable to escape the situation [4, 6]. According to Zych et al. [2], approximately one in three children is engaged in various types of bullying. Bullying can take many forms, like physical, verbal, or psychological harm, and can occur in various settings, such as in schools or online platforms [7–9]. The effects of bullying can be severe and long-lasting, causing physical and emotional harm, social isolation, and academic setbacks. Understanding the dynamic of bullying, its causes, and the roles that individuals play in perpetuating or preventing it is essential to developing effective strategies to address and prevent this harmful behavior [10].

In addition to the classic roles within bullying, like bully and victim, other roles have been identified, like defender, assistant, reinforcer, and outsider. Thus, each student in a class can be assigned a specific role [11]. The victim is defined as the person the bully targets, while the bully is defined as the active role engaging in aggressive or harmful behavior towards the victim. The defender is a role closely associated with the victim, he/she supports or protects the victim and takes efforts to actively intervene and stop bullying behaviors from occurring towards the victim. The assistant is the person helping the bully by participating in or encouraging their harmful behavior. It is also an active role, but rather than initiating bullying, the assistant follows the bully's behavior. Reinforcers also support the bully's behavior indirectly by laughing or watching like an audience without intervening. Lastly, the outsider is a person who is uninvolved in the bullying situation. It is important to note that individuals can occupy multiple roles within a bullying system and that these roles can shift and change over time [11]. Adolescents can occupy multiple roles simultaneously, and role scores are often moderately intercorrelated [11, 12]. However, prior factor analytic studies highlighted that the roles can be separated. Some studies also found pro-bully (e.g., bully, assistant, outsider) and pro-victim (e.g., victim, defender) roles [13, 14] while others did not [15, 16].

### School well-being

Student well-being is “a sustainable state characterized by predominantly positive feelings and attitude, positive relationships at school, resilience, self-optimization and a high level of satisfaction with learning experiences” [17]. It comprises positive and negative feelings and physical complaints in the school [17, 18], with positive feelings and attitudes summarizing positive affect [17]. Well-being encompasses the personal feelings schools evoke in the students [17]. In contrast, school climate includes different aspects such as: physical (i.e., the appearance of the building, size of the school, organizational aspects, resources, safety), social (i.e., relationships, fair treatment, competition between students, influence on decision making) and academic aspects (i.e., quality of lessons, expectations, monitoring of progress) [19]. Therefore, while these two constructs might be related, school well-being refers to the individual welfare of students, whereas school climate focuses on broader social and relational dynamics within the school [17, 19].

Keyes [18] proposed that students must show emotional, social, and psychological well-being to flourish. Negative affect is defined below as an emotional state of, for example, increased sadness, anxiety, anger, and boredom. In contrast, positive affect has been defined as being cheerful, proud, and happy at school [20]. Physical complaints encompass frequent head and stomach aches, tiredness, and nausea. Recurrent physical complaints that are accompanied by symptom-related psychological impairments can affect a child’s level of functioning and quality of life [21, 22]. Recent research has shown positive correlations between bullying, negative affect, and recurrent physical complaints [23–26]. Additionally, positive affect has been negatively associated with bullying victimization [25]. Additionally, Andreou et al. [27] were able to show that seriously bullied students report less school well-being than their peers who report less victimization. Studies also indicate that students who experience or witness bullying report lower levels of school well-being [28, 29].

### The moderating role of moral disengagement

Moral disengagement describes the process of disengaging from one's moral beliefs [30]. It also involves justifying aggressive and harmful behavior [30]. Bandura [31] distinguishes different disengagement practices like moral justification (e.g., justifying immoral behavior by a higher moral purpose), euphemistic labelling (e.g., using language to make immoral behavior seem less unethical or harmful), advantageous comparison (e.g., compare immoral behavior to someone else's worse behavior to make it seem more acceptable), displacement of responsibility (e.g., attribution of responsibility for immoral behavior to others, such as authorities), diffusion of responsibility (e.g., attribution of responsibility for immoral behavior to a group rather than taking personal response), disregard or distortion of consequences (e.g., justifying immoral behavior by minimizing or distorting the potential consequences of the immoral actions) and dehumanization (e.g., justifying immoral behavior by dehumanizing the victims, making it easier to mistreat). Studies have shown that moral disengagement, as part of moral cognition, can be associated with adopting roles within the bullying dynamic [12, 32–34]. Gini [12] found significant positive associations between the roles of assistant, reinforcer, bully, and moral disengagement. Significant negative associations between defender, outsider, and moral disengagement emerged, and no significant associations between the victim and moral disengagement [12]. Thornberg et al. [33] also found positive associations between moral disengagement and bullying and negative associations between moral disengagement and defending. Zych et al. [34], Georgiou et al. [35], and Montero-Carretero et al. [36] found positive significant associations between moral disengagement and bullying and victimization. In their meta-analysis, Killer et al. (2019) were able to identify significant associations between moral disengagement and bullying (positive), victimization (positive), and defending (negative). Bandura’s [30, 31] social-cognitive theory posits that disengaging moral self-sanctions reduces the emotional costs of harmful conduct. Applied to the school context, moral disengagement may buffer perpetrators and assistants from guilt or social anxiety, sustaining their school well-being despite aggressive behavior. Conversely, disengagement may be less relevant or even maladaptive for victims or defenders, as their well-being is more tied to peer support and fairness perceptions.

### Current study

Previous research suggests that adolescents involved in bullying are at increased risk of developing physical complaints and negative affect [23–26]. Mùzquiz et al. [25] also show that positive affect is negatively associated with bullying victimization. Additionally, moral disengagement has been linked to bullying [37–39] and subjective well-being [40, 41]. There seems to be a research gap for moral disengagement and school well-being. To extend the current body of research and explore the bullying-dynamic in different roles, the present study examines whether moral disengagement acts as a moderator variable between school well-being and the bullying roles.

Although bullying roles are interrelated [11], prior research demonstrates that they can differ substantially in their psychosocial correlates and well-being outcomes [27, 33]. Additionally, the roles are empirically distinct factors [15, 16]. Testing moderation separately allows for role-specific patterns to emerge without suppressor effects from multicollinearity among roles, which tend to be moderately to highly intercorrelated [12]. Given these theoretical distinctions [30, 31] and empirical overlaps [11], we test moderation models separately for each role to disentangle unique effects while reporting intercorrelations among roles to contextualize findings.

We hypothesize that: H1: All five bullying roles will show negative associations with school well-being, reflecting prior evidence of emotional distress and physical complaints among involved students [23, 27].

H2: Moral disengagement will moderate these associations. Specifically, higher disengagement will weaken the negative link to well-being for bully, assistant, and outsider roles, consistent with Bandura’s [30, 31] theory of self-sanction regulation. For victims and defenders, moral disengagement is expected to show little or no buffering effect, as their distress and prosocial motives are less dependent on moral justifications.

Understanding whether moral disengagement weakens the negative link between bullying involvement and school well-being provides insight into potential cognitive coping strategies. If disengagement protects well-being in some roles but not others, interventions can be tailored, for example, addressing maladaptive justifications among perpetrators while strengthening adaptive coping among victims. Although we did not expect moral disengagement to buffer school well-being for victims or defenders substantially, we included these roles for two reasons: (a) prior findings are mixed. Some studies report small but significant associations between moral disengagement and victimization [32, 34], suggesting that even victims may occasionally rationalize or normalize harmful experiences to maintain belonging; and (b) defenders may experience moral strain when standing up for peers, and disengagement could theoretically protect their well-being by reducing empathic distress. Testing these roles ensured a comprehensive examination of all roles within the bullying dynamic. It allowed us to verify whether the absence of moderation effects for these groups could be empirically supported.

## Method

### Participants

216 (53.7% female) secondary school students from Bremen and Lower Saxony, Germany, participated in the study. They had a mean age of 12.84 years (*SD* = 1.39 years). A power analysis was conducted to determine if the study sample had sufficient power. The a priori test showed that a sample size of *N* = 74 was necessary to detect a medium effect size (*f*^*2*^ = 0.15, α err prob = 0.05, power (1-β err prob) = 0.95) using a linear multiple regression, fixed model, single regression coefficient, with two predictors. [42, 43].

### Procedure

The Commission for Research Assessment and Ethics and the state education authority approved the project. The school administration of nine secondary schools in Bremen and Lower Saxony, Germany, decided to participate in the studies. After the approval of the schools was obtained, students were given consent forms to sign. Only students whose parents signed the forms were allowed to participate. Data collection occurred from January 2022 until July 2022 and was processed pseudonymously.

## Measures

### School well-being

School well-being was assessed using scales from the Bielefelder Longitudinal Study on Learning in Inclusive and Exclusive environments [44]. The scale consists of 11 Items asking students to indicate how often they experienced physical complaints (e.g. “How often do you have headaches at school?”—recoded; 4 Items) from never (0) to always (3) and how strongly they agreed with statements regarding their emotions at school (e.g. “Have you been cheerful a lot in school this past week?”; 7 Items) from disagree (0) to agree (3). Schwinger et al. (2015) provide evidence for the scores' factorial, convergent, and divergent validity. For this study, the scales' negative affect and physical complaints were reverse-scored and added with the positive affect score to depict school well-being.

### Bully participant behaviour questionnaire

The different bullying roles were measured using the *Bullying Participant Questionnaire* (BPBQ; Summers & Demaray, 2008). The BPBQ is a self-report measure in which students were asked to answer questions regarding how often they engage in certain behaviors in the last 30 days (0 = never, 1 = 1 to 2 times, 2 = 3 to 4 times, 3 = 5 to 6 times, 4 = 7 or more times). Each role (Bully, Victim, Assistant, Defender, and Outsider) consists of 10 Items (e.g., Bully: “I have called another student bad names”; Victim: “I have been called mean names “; Assistant: “I have made fun of someone when they were pushed, punched, or slapped”; Defender: “I helped someone who was purposely tripped”; Outsider: “I pretended not to notice when someone else tripped another student on purpose”). The roles are captured in their manifestation (sum scores) and not as categories. To avoid priming the students, no definition of bullying was given [45–48]. The questions are designed to contain the characteristics of bullying behaviors. The scores for each role were calculated by adding the items. Demaray et al. [49] provide evidence for the scores' congruent, convergent, and divergent validity. Students could have high means in more than one of the roles.

### Moral disengagement

The questionnaire developed by Ribeaud and Eisner [50, 51] was used to evaluate moral disengagement. It focuses on justifying/neutralizing aggressive behavior and bullying. The questionnaire contains 18 items, such as "It is acceptable to fight to defend your friends.". They were asked to indicate how strongly they agree with the statements from strongly disagree (0) to strongly agree (3). Individual scores were obtained by adding the responses to each question. Ribeaud and Eisner [50, 51] report construct and criterion validity for their questionnaire.

### Data analysis

To investigate the moderation effects of moral disengagement on the association between bullying and school well-being, a series of multiple linear regression models was conducted using the statistical software R. Patterns of missingness were examined using Little’s MCAR test [52]. Results indicated that data were consistent with being Missing Completely At Random (MCAR), χ^2^(157) = 161.0, *p* = 0.398. Because the assumption of MCAR was met, missing values were imputed using predictive mean matching (PMM) [53, 54]. This approach preserves the distributional properties of the data and yields plausible values by drawing from observed cases with similar predicted values.

For each role variable (bully, assistant, outsider, victim, defender), an interaction term was created by centering both the role variables and moral disengagement and then computing their product term to test the moderation hypothesis. Separate moderation models were estimated with school well-being as the dependent variable, regressed on the centered role variable, centered moral disengagement, and their interaction term.

All models were checked for compliance with regression assumptions. Normality of residuals was examined visually via Q-Q plots and tested using the Shapiro–Wilk test. Residuals deviated somewhat from normality (all *p* < 0.01) [55]. Due to the bootstrapping process, the non-fulfillment of the normality assumption is not problematic [56]. Homoscedasticity was assessed with residuals vs. fitted plots and the studentized Breusch-Pagan test. All models showed no indication of heteroscedasticity (all *p* > 0.62) [57]. Multicollinearity was tested with variance inflation factors (VIF). Across all models, VIF values ranged from 1.00 to 1.99, well below the common threshold of 10, indicating no problematic collinearity [58].

To obtain robust estimates of regression coefficients, we performed nonparametric bootstrapping with 2000 resamples for each model. Percentile-based 95% confidence intervals were calculated for all predictors, including the interaction terms.

Because five separate moderation models were estimated, we controlled for multiple testing of the interaction terms. Raw *p*-values were extracted for the interaction effects and adjusted using the Holm method [59]. We made the corrections based on the hypothesis, so there was one correction for the victim roles and one for the bully roles [59]. The R code from the analysis can be obtained from: https://osf.io/8z3h4/?view_only=40290cb918184961970401c0f49ecc48.

## Results

Table 1 presents sample size, means, standard deviations, and Cronbach's alpha coefficients for all scales used in the study. The mean of the defenders is the highest, followed by victims, bullies, outsiders, and assistants. The Cronbach's alpha values are satisfactory.

**Table 1:** Descriptive statistics of the variables

Variable	N	M	SD	α
Bully^1^	208	5.10	5.43	.82
Victim^1^	210	7.95	8.15	.90
Defender^1^	212	8.57	8.07	.92
Assistant^1^	211	2.50	3.70	.78
Outsider^1^	207	4.69	6.86	.91
Moral disengagement^2^	203	10.59	7.85	.88
School well-being^3^	175	21.29	5.56	.83

*M = *Mean, *SD = *Standard deviation, *α* = Cronbach's alpha, higher M indicates higher involvement in the respective role, higher moral disengagement and higher school well-being, we used sum scores, ^1^10 Items (0 = never, 1 = 1 to 2 times, 2 = 3 to 4 times, 3 = 5 to 6 times, 4 = 7 or more times) ^2^18 Items (0 = strongly disagree to 3 = strongly agree), ^3^11 Items (0 = never/disagree to 3 = always/agree)

Table 2 indicates high correlations between the bullying roles, except for the defender, which does not correlate with the perpetrator roles. Additionally, moral disengagement correlates with all variables except the defender. School well-being negatively correlates with the Bully, Victim, Assistant, and Outsider.

**Table 2:** Correlations between the used variables

	1	2	3	4	5	6	7
1.Bully	-	.52^***^	.13	.76^***^	.43^***^	.51^***^	-.24^***^
2.Victim		-	.55^***^	.51^***^	.32^***^	.33^***^	-.38^***^
3.Defender			-	.09	-.07	.01	-.08
4.Assistant				-	.59^***^	.46^***^	-.16^*^
5.Outsider					-	.44^***^	-.17^*^
6.Moral disengagement						-	-.11
7.School well-being							-

^*^p <.05; **p <.01; ***p <.001

A moderation analysis was performed for each role to determine if the interaction between the bullying role (bully, victim, defender, assistant, outsider) and moral disengagement is significantly associated with school well-being. The assumptions for each model were checked and are reported in Table 3.

**Table 3:** Assumption checks and regression weights

Role	Predictor	B (SE)	95% CI	t	p	Adjusted p	Shapiro–Wilk	Breusch-Pagan	VIF
Bully	Bully	-.29 (.10)	[-.50; -.12]	−2.87	.004		W =.98 *p* <.001	BP =.11, *p* =.99	1.96
	Moral Disengagement	.02 (.06)	[-.08;.13]	0.35	.725				1.34
	Interaction	.01 (.01)	[-.00;.03]	1.56	.119	.359			1.71
Assistant	Assistant	-.26 (.14)	[-.56; -.04]	−1.79	.075		W =.98, *p* =.002	BP = 1.75, *p* =.626	1.89
	Moral Disengagement	-.01 (.06)	[-.13;.09]	−0.26	.794				1.29
	Interaction	.01 (.01)	[-.01;.03]	1.16	.246	.362			1.76
Outsider	Outsider	-.24 (.08)	[-.39; -.09]	−2.95	.003		W =.98, *p* =.003	BP =.26; *p* =.967	1.99
	Moral Disengagement	.02 (.05)	[-.08;.11]	0.43	.669				1.22
	Interaction	.01 (.01)	[-.01;.02]	1.34	.181	.362			1.75
Victim	Victim	-.26 (.05)	[-.35; -.18]	−5.36	<.001		W =.97; *p* <.001	BP = 1.62, *p* =.654	1.19
	Moral Disengagement	.01 (.05)	[-.10;.11]	0.22	.820				1.20
	Interaction	.01 (.01)	[.00;.02]	2.42	.016	.033			1.19
Defender	Defender	-.02 (.05)	[-.10,.07]	−0.41	.682		W = 0.98; *p* =.002	BP = 0.81; *p* =.847	1.01
	Moral Disengagement	-.04 (.05)	[-.15;.05]	−0.88	.379				1.00
	Interaction	.01 (.01)	[-.00;.02]	1.16	.246	.246			1.01

The bully model explained 2.7% of variance in school well-being, F(3, 212) = 2.98, *p* = 0.032. Bullying was negatively associated with school well-being (β = −0.29, *p* = 0.004, 95% CI [−0.50, −0.12]). Moral disengagement showed no significant association (β = 0.02, *p* = 0.725, 95% CI [−0.08, 0.13]). The interaction of bullying and moral disengagement was insignificant (β = 0.01, *p* = 0.120, 95% CI [−0.003, 0.027]; Holm-adjusted *p* = 0.359).

The assistant model explained 0.4% of variance, F(3, 212) = 1.81, *p* = 0.146. The main effect of assistant behavior was marginally negative (β = −0.26, *p* = 0.075, 95% CI [−0.56, −0.04]), but moral disengagement was unrelated to school well-being (β = −0.01, *p* = 0.794, 95% CI [−0.13, 0.09]). The assistant and moral disengagement interaction was nonsignificant (β = 0.01, *p* = 0.246, 95% CI [−0.006, 0.034]; Holm-adjusted *p* = 0.362).

Regarding the outsider, the model explained 3.0% of variance, F(3, 212) = 3.04, *p* = 0.030. Outsider behavior was negatively associated with school well-being (β = −0.24, *p* = 0.004, 95% CI [−0.39, −0.09]), whereas moral disengagement was not significant (β = 0.02, *p* = 0.669, 95% CI [−0.08, 0.11]). The outsider and moral disengagement interaction was nonsignificant (β = 0.01, *p* = 0.181, 95% CI [−0.008, 0.018]; Holm-adjusted *p* = 0.362).

The defender model explained 0% of variance, F(3, 212) = 1.02, *p* = 0.384. Defender behavior was not significantly associated with school well-being (β = −0.02, *p* = 0.682, 95% CI [−0.10, 0.07]), and moral disengagement was likewise nonsignificant (β = −0.04, *p* = 0.379, 95% CI [–0.15, 0.05]). The defender × moral disengagement interaction was nonsignificant (β = 0.01, *p* = 0.246, 95% CI [−0.003, 0.020]; Holm-adjusted *p* = 0.246).

**Fig. 1:**
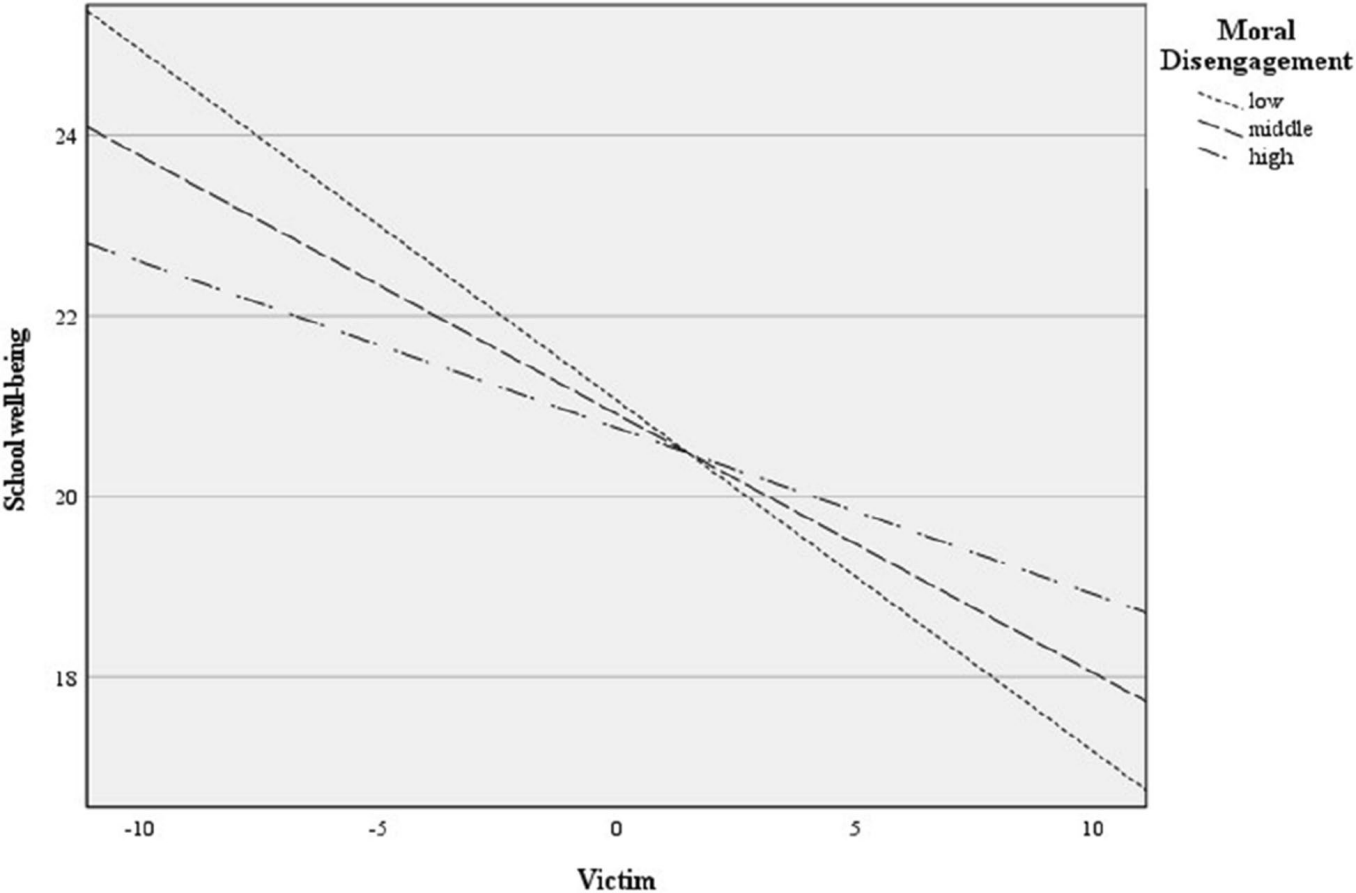
Moderation analysis

Only the model with the interaction between victim and moral disengagement significantly predicted school well-being (see Fig. 1). The overall model explained 11.5% of variance, F(3, 212) = 8.91, *p* < 0.001. Victimization was strongly and negatively associated with school well-being (β = −0.26, *p* < 0.001, 95% CI [−0.35, −0.18]). Moral disengagement showed no significant effect (β = 0.01, *p* = 0.830, 95% CI [−0.10, 0.11]). The victim × moral disengagement interaction was significant (β = 0.01, *p* = 0.016, 95% CI [0.004, 0.022]), and this effect remained significant after Holm correction (*p* = 0.033). A more detailed analysis using Johnson-Neyman calculation [60] indicated that the moderation effect of victim and moral disengagement on school well-being was significant at all the levels (low (*ß* = −0.36 SE = 0.05, *t*(1, 16) = −5.10, *p* < 0.001), middle (*ß* = −0.26, *t*(1, 16) = −5.36, *p* < 0.001) and high (*ß* = −0.16, *t*(1, 16) = −2.92, *p* < 0.001) levels of moral disengagement). The statistical significance transition point was z = 10.54. In other words, victimization negatively predicted school well-being primarily at moderately high levels of moral disengagement, while at lower levels the effect was not significant. This pattern supports a moderation effect whereby moral disengagement amplifies the association between victimization and diminished school well-being.

## Discussion

The results show that moral disengagement is positively correlated with the bullying roles of victim, bully, assistant, and outsider. The more the students got involved in the bullying dynamics, the more they disengaged from moral standards (see also Siddiqui et al., 2025). This is nearly consistent with the meta-analysis of Killer et al. [32]. However, Killer et al. [32] found no correlations between outsiders and moral disengagement. Furthermore, moral disengagement was not correlated with the defender role in our study, even though the meta-analysis showed effects. This could be due to the different instruments (global or differentiated in forms of bullying, e.g., physical, verbal, relational) and survey procedure (peer nomination and self-report) used in the meta-analysis and our study. The analysis of moderating variables revealed significant differences in the associations between moral disengagement and the bullying roles regarding self-report and peer nomination (bully and victim) or the ratio of females (defender) [32]. The non-significant correlation between defending and moral disengagement might therefore be linked to the current study's sample. Additionally, the significant correlation between moral disengagement and the role of the outsider might not be in line with the meta-analysis by Killer et al. [32]; however, it does coincide with other studies [61, 62].

Additionally, the bullying roles victim, bully, assistant, and outsider correlated negatively with school well-being. Beyond these correlational findings, the moderation analyses revealed important direct effects that align with theoretical frameworks. Bullying behavior showed a significant negative association with school well-being, consistent with social-ecological theory, which posits that aggressive behaviors create hostile school environments that undermine students’ sense of safety and belonging [63]. Similarly, outsider behavior was negatively associated with school well-being, supporting bystander theory’s assertion that passive witnessing of aggression can create psychological distress and feelings of helplessness [64]. The moderation analysis further revealed that victimization showed the most substantial adverse effect on school well-being. Aligning with stress and coping theory, which emphasizes how chronic peer victimization depletes psychological resources and disrupts adaptive functioning in academic settings [65]. This is consistent with studies reporting that children and adolescents involved in bullying dynamics report more anxiety and depression [66], more negative affect [23, 25], recurrent physical complaints [24, 26], and subjective well-being [40, 41]. The defender role and moral disengagement did not correlate with school well-being. The insignificant association between defending and school well-being is not in line with the results of current studies examining school climate and defending [67]. Additionally, studies investigating the association between moral disengagement and school climate found significant negative links between the two variables [36], which is also not in line with our findings. However, we examined school well-being and not school climate; therefore, the associations could be different, although there is some overlap in the questions asked regarding school well-being and school climate.

Against our hypothesis, the moderation analysis only revealed significant associations for the victim role. Moral disengagement did not moderate the association between school well-being and the other bullying roles (bully, defender, outsider, and assistant). The results of the significant moderation analysis revealed that the more experience one has as a victim, the worse they feel in school. However, the results also show that detachment from moral standards reduces this connection. Moral disengagement may be a coping strategy, but our cross-sectional design precludes causal claims. We therefore interpret this possibility cautiously. It can be assumed that students who experience bullying disengage or diminish the importance of moral standards.

Huesmann's [68] theory of scripts could be an explanatory approach for the identified moderating effect. Cognitive scripts are mental structures or frameworks that organize and guide understanding of specific situations, events, or actions. They help individuals to respond to challenging situations by guiding them on what they should do and what is expected. The theory of social information processing by Crick and Dodge [69], Lemerise and Arsenio [70], and Garrigan et al. [71] is a theoretical perspective explaining aggressive behavior and how individuals interpret and respond to social information. The internalized scripts are stored in the database and may persist, reinforcing the internalization of the scripts every time they are enacted. One possible interpretation, which remains speculative given our cross-sectional data, is that repeated victimization may be associated with the development of distorted cognitive scripts or perceptions. However, our data do not allow for causal conclusions, and longitudinal research would be necessary to test this pathway. To cope with this situation, strategies of moral disengagement are used, such as normalizing victimization or dehumanizing themselves.

Another possible explanation could be that students place less value on moral standards, as others do not adhere to the rules set by society, in victimizing the students. Some psychoanalytic perspectives, such as perpetrator introjection [72, 73], have been proposed to explain victims’ internalization of aggressor attitudes. While potentially thought-provoking, these perspectives were not empirically tested here and are mentioned only as one of several possible frameworks for future research. Perpetrator introjections are internal psychological parts of a person that the victim manifests. In research, introjection usually occurs after traumatic experiences such as physical violence. Individuals who have had such experiences may try to protect themselves by distancing themselves from their own feelings and thoughts and instead adopt internalized representations, thoughts, emotions and behavior of the perpetrator and accept it as part of their own self—lessening the importance of moral standards as others do not adhere to these standards [72–75]. This can allow the victim to attempt to control and protect themselves by denying and suppressing their own vulnerability. Perpetrator introjects can be an unconscious survival mechanism to cope with the traumatic experiences that turn into a conscious defense mechanism [73]. Howell [73] describes this mechanism as follows: “The weak and undeveloped personality reacts to sudden unpleasure not by defense, but by anxiety-ridden identification and introjection of the menacing person or aggressor”.

Both mechanisms (scripts and introjections) may also be applied to bullying dynamics. The victims adopt the thoughts and feelings of the bully. Students who experience intense bullying feel uncomfortable at school as a result. To cope with the situation, they detach themselves from their moral values such as fairness, justice, or integrity. They adopt the thoughts and feelings of the bully. They may think they deserve the harmful actions that serve them right. This internalization process may also contribute to the victim's continued perception of himself as part of the community and his desire to maintain the relationship with the bully and the class [75, 76]. Bandura’s social-cognitive theory of moral disengagement [30, 31] helps explain how disengagement might buffer the emotional costs of harmful behavior. However, Bandura primarily developed this model to describe perpetrators’ justification of aggressive acts. Applying it to victims, as a coping mechanism for repeated harm, extends the framework beyond its original scope and should be treated as exploratory rather than confirmatory. Our findings suggest associations compatible with this idea but do not directly validate or extend Bandura’s theory.

Despite estimating separate models, the absence of moderation effects for bully, assistant, outsider, and defender roles suggests that the buffering function of moral disengagement may not generalize across roles. For perpetration-proximal roles, prior work consistently links higher moral disengagement to greater involvement in bullying [30, 31, 33], and meta-analytic evidence indicates robust positive associations with perpetration and negative associations with defending [32]. If disengagement is already elevated among bullies and assistants, restricted variance may limit its capacity to attenuate the association with school well-being further, yielding null interactions even when main-effect associations are present. For outsiders, findings are mixed; the meta-analysis by Killer et al. [32] did not identify a reliable association between outsider behavior and moral disengagement, which makes an interaction with well-being less plausible. For defenders, studies typically report lower disengagement and negative links with disengagement [32, 33]. This is conceptually inconsistent with a buffering mechanism and may again reduce the statistical room for interaction effects. Methodological features likely contributed as well. Our role indicators are self-reports, and we used a global moral disengagement scale rather than role- or context-specific disengagement; meta-analytic work shows that associations between disengagement and bullying roles vary by measurement approach and informant source [32]. Moreover, our outcome captures school well-being rather than school climate; studies linking disengagement to climate report negative associations at the contextual level [36], which may not translate into role-specific buffering of individual well-being in cross-sectional data. These considerations suggest that the null findings do not contradict social-cognitive theory but indicate that disengagement’s role is more consistent as a correlate of role involvement than as a moderator of the role–well-being link outside victimization. Replication with larger samples, multi-informant role assessments, and more granular disengagement measures is needed to test these role-differentiated expectations [11, 12, 25, 27].

Because our data are cross-sectional and based on self-reports, any directional or causal statements remain speculative. Our findings should be understood as associations, and future longitudinal or experimental designs are required to establish temporal order or causality.

### Limitations and further research

One potential limitation is that the used ad hoc sample may not be representative of the population, which may introduce bias and limit the generalizability of findings. Similarly, the used cross-sectional design may not provide a complete understanding of the phenomenon and associations between victimization, moral disengagement, and school well-being. The results are limited in their ability to establish causality or examine changes over time, as they only capture data from a single moment. Future studies should investigate the longitudinal associations between the variables. Additionally, the models of bullying roles (bully, assistant, outsider, and defender) did not show significant moderating effects. This may be due to the design or the low sample size. The sample size in this study is not large enough to attribute only one role to the students, leading to high correlations between the roles. Therefore, future studies should examine the moderating effects with samples that can be categorized as occupying only one of the bullying roles. Additionally, studies should focus on the internalized scripts, representations of the self-concept, and self-efficacy of students to investigate whether these are used as coping strategies in bullying situations. Because all measures were self-reported and conceptually related, common method variance and construct overlap cannot be entirely ruled out. Although this is a common challenge in bullying research, future work should incorporate multi-informant approaches (e.g., peer nominations, teacher reports) or latent-variable modeling to reduce shared-method bias. The intercorrelations among roles (see Table 2) suggest overlapping experiences within bullying dynamics. While this reflects the fluid nature of roles in real-world settings, it also increases the risk of multicollinearity. We therefore report intercorrelations and emphasize caution in interpreting role-specific effects. Given moderate to high correlations and our limited sample size, we opted for separate moderation models to minimize multicollinearity artifacts but acknowledge the increased family-wise error and reduced power this entails. Testing five separate moderation models in a modest sample (*N* = 216) may stretch the data and limit statistical power. We chose separate models to avoid suppressor effects and to allow role-specific patterns to emerge. To mitigate Type I error inflation, we used centered variables, reported exact p-values, and interpreted results conservatively. Future studies should replicate these findings with larger samples and employ multivariate approaches (e.g., structural equation modeling or multilevel modeling with latent constructs) when the sample size does not support multiple tests.

### Practical implications and conclusion

Repeated victimization experiences negatively influence the self-concept, self-efficacy, and school well-being of students. Additionally, the situation can add to the manifestation of negative scripts and negative self-perception. In order to break the cycle of repeated victimization, students' self-efficacy needs to be increased. Students also need to be provided with opportunities to escape repeated victimization. Furthermore, schools and teachers need to highlight that victimization is not normal. A teacher could help students develop adaptive cognitive scripts that promote positive self-perceptions. Attitudes of teachers regarding bullying should also be investigated (i.e., normative beliefs need to be addressed), and teachers should be provided with information to spot bullying and opportunities to learn how to intervene in these situations effectively. Additionally, the school should create a safe and supportive school environment. By addressing negative scripts and perpetrator introjections and promoting positive self-perceptions and social interactions, teachers and school practitioners can help improve the overall school well-being and reduce victimization.

## Data Availability

The data that support the findings of this study are available from the corresponding author upon reasonable request. The data are not publicly available due to privacy or ethical restrictions.

## Abbreviations

BPBQ: Bully Participant behavior Questionnaire
MCAR: Missing completely at random
PMM: Predictive mean matching
VIF: Variance inflation factors
CI: Confidence interval
